# 4SALE – A tool for synchronous RNA sequence and secondary structure alignment and editing

**DOI:** 10.1186/1471-2105-7-498

**Published:** 2006-11-13

**Authors:** Philipp N Seibel, Tobias Müller, Thomas Dandekar, Jörg Schultz, Matthias Wolf

**Affiliations:** 1 Department of Bioinformatics, Biocenter, University of Würzburg, Am Hubland, Würzburg, Germany

## Abstract

**Background:**

In sequence analysis the multiple alignment builds the fundament of all proceeding analyses. Errors in an alignment could strongly influence all succeeding analyses and therefore could lead to wrong predictions. Hand-crafted and hand-improved alignments are necessary and meanwhile good common practice. For RNA sequences often the primary sequence as well as a secondary structure consensus is well known, e.g., the cloverleaf structure of the t-RNA. Recently, some alignment editors are proposed that are able to include and model both kinds of information. However, with the advent of a large amount of reliable RNA sequences together with their solved secondary structures (available from e.g. the ITS2 Database), we are faced with the problem to handle sequences and their associated secondary structures synchronously.

**Results:**

4SALE fills this gap. The application allows a fast sequence and synchronous secondary structure alignment for large data sets and for the first time synchronous manual editing of aligned sequences and their secondary structures. This study describes an algorithm for the synchronous alignment of sequences and their associated secondary structures as well as the main features of 4SALE used for further analyses and editing. 4SALE builds an optimal and unique starting point for every RNA sequence and structure analysis.

**Conclusion:**

4SALE, which provides an user-friendly and intuitive interface, is a comprehensive toolbox for RNA analysis based on sequence and secondary structure information. The program connects sequence and structure databases like the ITS2 Database to phylogeny programs as for example the CBCAnalyzer. 4SALE is written in JAVA and therefore platform independent. The software is freely available and distributed from the website at

## Background

Since multiple sequence alignments are the basis of many analyses, for example in phylogenetics or in analysing functional protein domains, there is a need for programs to create and improve those alignments. Currently, several programs are available to fulfil these necessities, e.g., CLUSTAL W [1], MUSCLE [2], DiAlign [3], T-Coffee [4] or DCA [5] all of which are able to align multiple sequences globally. The underlying methods have their strengths and weaknesses, and resulting alignments can diverge from the biologically correct ones. Editors like JalView [6], SEAVIEW [7], CINEMA [8] or Align [9] are needed to enhance the results by hand.

The just mentioned tools are based on sequence information only, but in RNA sequence analyses there is often also structural information available. Databases like the ITS2 Database [10-12] provide a growing number of sequences and their known secondary structures, as a prerequisite for constructing RNA alignments for inferring phylogenies, which of course is a precondition to understand the evolution of such RNA secondary structures [12].

All available methods that include structural information to build RNA sequence alignments have a very high complexity. Rfam [13] provides a method to compare a single nucleotide query sequence to handcurated alignments of non-coding RNA families with annotated consensus secondary structures. MARNA [14] and RNAforester [15] can be used to build global multiple alignments based on sequence and simultaneously on secondary structure information. However, the amount of sequences and/or sequence lengths is limited due to the complexity of their underlying algorithms, which is at least *O*(*N*^3^).

In current alignment editors like RALEE [16], DCSE [17] or jPHYDIT [18] secondary structure information support is very limited. While RALEE relys on the consensus structure only, jPHYDIT just shows the pairing information of the selected sequence. Another RNA alignment editor called SARSE has become available recently and focuses on detection and editing of structural groups in RNA families [19]. So there is no editor available to align both, sequence and secondary structure information of every single RNA sequence simultaneously.

## Implementation

4SALE is entirely written in JAVA, which enables to execute the software on any platform with a JAVA 5.0 virtual machine available. The application consists of two parts, the alignment algorithm, which is based on standard protein alignment algorithms, and the graphical editor frontend. For sequence and secondary structure alignments running on the local machine 4SALE takes use of CLUSTAL W [1], so the binary is required to be installed.

Integration of different multiple alignment tools is realised by using SOAP based WebServices. Here, we take use of RNAforester [15], CLUSTAL W [1], DCA [5] or DiAlign [3]. The DiAlign and DCA WebServices currently support sequence alignments without secondary structure information only. The WebService technology enables the user to run the tasks on remote machines. Therefore, it is possible to use 4SALE without restriction of any kind during the calculation of the alignment. All WebServices require an internet connection.

## Results

### Supported data

For RNA sequence and secondary structure alignment and editing, 4SALE reads Vienna style DotBracket [20] formatted files. The ITS2 Database [10-12] represents a good source for these kind of data. In addition we provide direct access to the ITS2 Database from within 4SALE through the ITS2 SOAP interface [10]. While RNA sequence information only is supported via the standard FASTA format, alignment data can be loaded using the Clustal [1] importer. Furthermore 4SALE handles XML based RNA formats namely RNAStructML and RNAStructAlignmentML [21].

### Standard features

In addition to the secondary structure based functionality, 4SALE integrates many useful features, that are known from other alignment editors/programs. This includes selecting multiple sections of an alignment to highlight interesting regions and temporarily hide sequences to focus on a subset of the alignment. Sequence-motifs, including those, which are based on sequence and secondary structure information, can easily be highlighted by pattern matching. Alignment column conservation based on sequence information is visualized by either sequence logos [22] or simply by bars on top of each column. Further importing, exporting and deleting sequences is possible by using the sequence names' context menu. Additionally sequences can be reordered with the help of the "Rearrange Sequences" window.

### Algorithm

**Figure 1:**
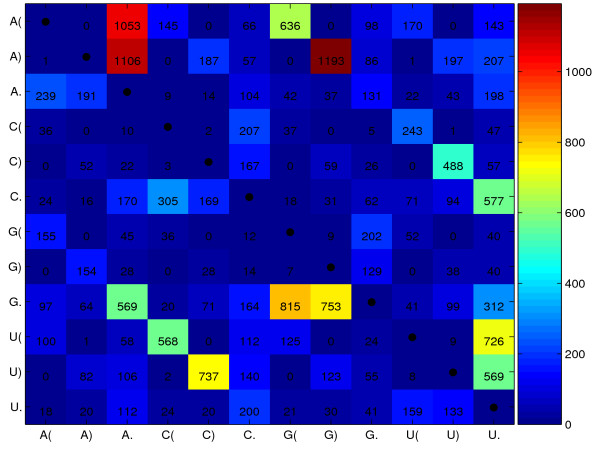
**ITS2 sequence and secondary structure ratematrix**. This figure shows the estimated sequence/secondary structure substitution rates (*10^5^). Diagonal entries are by definition the negative sum of all row entries. Note, high rates depict frequent substitutions, and vice versa small rates depict rare substitutions, e.g., within a secondary structure Cs an Us are often replaced by each other.

Beside the integration of RNAforester [15] we developed an algorithm that uses the secondary structure information of every single sequence to align multiple RNA sequences. This algorihm inherits the complexity of those based on sequence information only. We achieve this by mapping the sequence and secondary structure information of every single RNA sequence to artificial protein sequences. The algorithm can be described as string alignment on a 12 letter alphabet comprised of the 4 nucleotides in three structural states (unpaired, paired left, paired right). Horizontal dependencies given by the sequence bindings are not modeled by this approach. To align the string we use common alignment programs, like CLUSTAL W [1] with a suitable scoring matrix. There are several substitution models for this kind of scoring matrices discussed [23,24], we used a model as described by [25,26]. The model is based on subsitutions that were extracted from ITS2 sequence and secondary structure alignments (Fig. 1). Those sequences and their associated secondary structures were obtained from the ITS2 Database [10-12].

### Synchronous editing

One of the main features of 4SALE is synchronizing the sequence and secondary structure alignment, that is, every operation on the sequence alignment is also performed on the secondary structure alignment and vice versa. Alignment editing in general works like in most alignment editors by using the space key to insert and the backspace key to remove gaps.

#### Block editing

**Figure 2:**
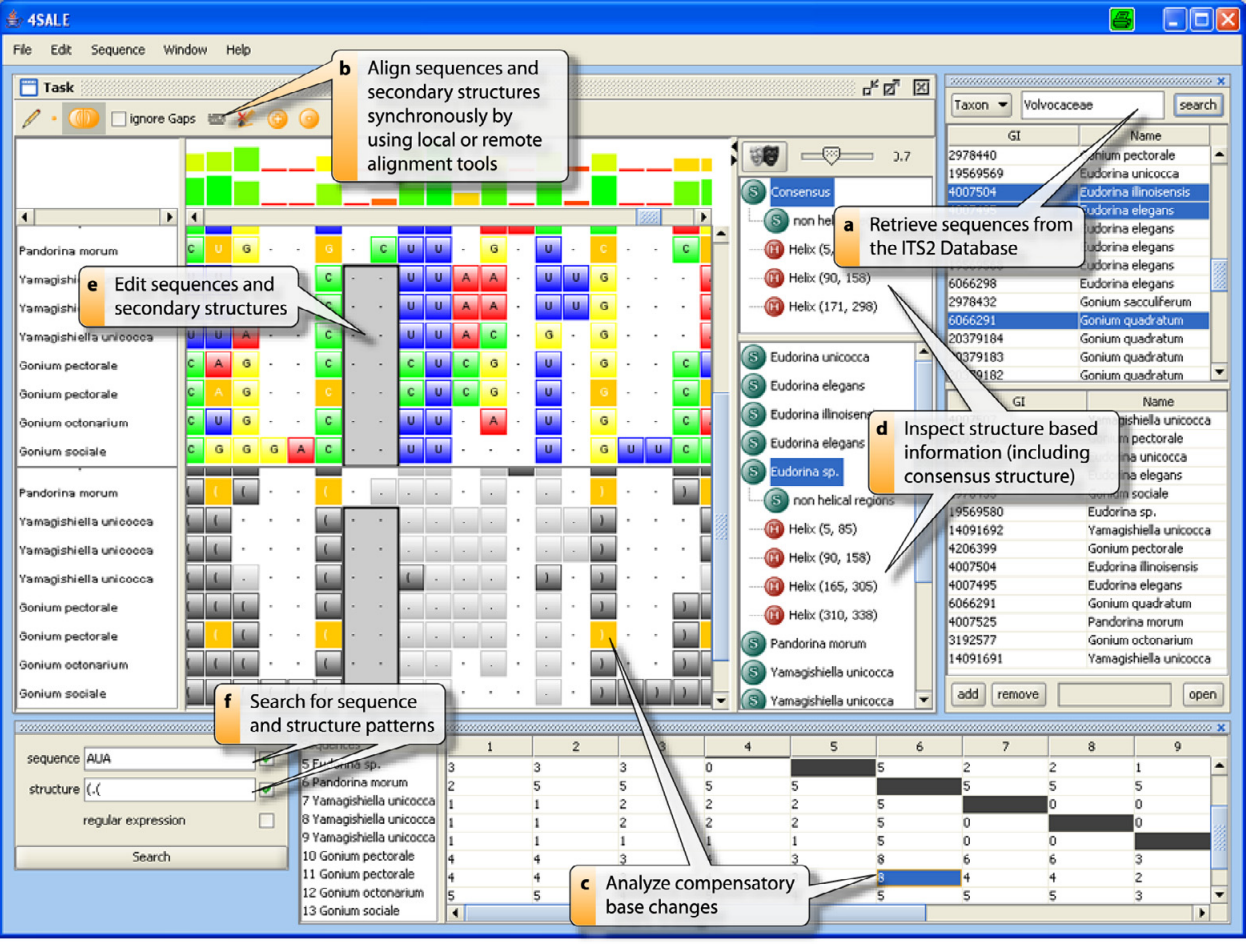
**Overview**. This figure shows a complete overview of the main features in 4SALE. All parts and their use are described in the boxes within the figure.

As an effect of not using horizontal dependencies in our greedy sequence and secondary structure alignment algorithm, there are often misaligned "blocks" in the result of CLUSTAL W [1]. As shown in Fig. 2e the alignment could be improved very fast, by using the block editing feature in 4SALE.

#### Cursors

To make editing as convenient as possible, we provide different edit cursors. Beside the standard cursor, that behaves as you expect from text editors for example, we have an exclusive cursor, which performs the edit operation on every sequence but the selected ones. The leftside cursor (c.f. [9]) allows edit operations only at the beginning of the sequence. As mentioned above all cursors perform synchronously on sequences and their secondary structures.

### Working with secondary structures

As current predictions of secondary structure information is not highly reliable, performing changes to correct the secondary structures is often needed.

#### Adding secondary structure information

4SALE supports two methods to add secondary structure information to RNA sequences. First, by using the remote folding feature to call the RNAfold WebService provided by the University of Bielefeld (Germany), or manually adding secondary structures by using the secondary structure editing feature.

#### Secondary structure editing

The secondary structure editing mode available in 4SALE allows easy modification of the secondary structure information. It is context sensitive, which means it uses sequence and secondary structure information to validate whether a binding in this context is possible or not. Furthermore, it supports column based editing by holding the control key. This enables insertion and deletion of equivalent bindings in all sequences. In this case bindings are only added to sequences allowing this binding (same with deletions).

### Secondary structure inspector

A secondary structure inspector allows to view and select specific helical regions in secondary structures of loaded sequences. The inspector consists of two parts; the upper part shows a consensus of all secondary structures, the lower part shows all secondary structures separately. The secondary structure consensus is calculated not only on column conservation, but also with respect of horizontal dependencies, so the result is a valid secondary structure. The conservation threshold can be modified using the slider above.

**Figure 3:**
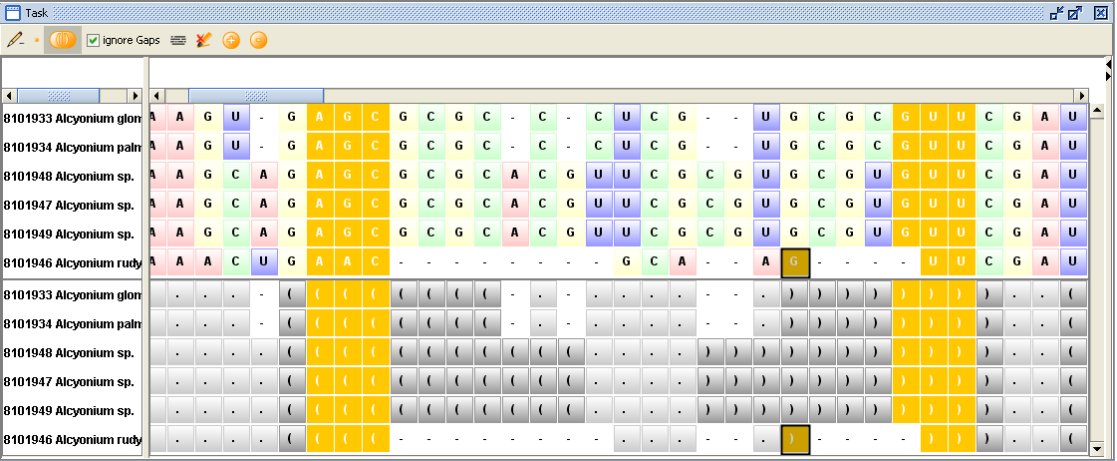
**Synchronous editing**. This figure illustrates the synchronous sequence and secondary structure handling in 4SALE. When selecting a helical region in the secondary structure alignment, as shown in this example, 4SALE synchronously selects its structural counterpart and its corresponding parts in the sequence alignment. The figure also shows very well, how easily an error in the alignment could be detected and corrected by using the selection and edit features in 4SALE.

Selecting an element in the inspector highlights the corresponding part in the alignment view. As shown in Fig. 3, the inspector simplifies visualizing misaligned sequences. "Masking" the sequence alignment based on the current consensus structure is also possible. The result is shown in a new window, which contains the alignment based on sequence information only. The alignment can be processed like any other sequence alignment loaded in 4SALE. This is particularly useful for calculating phylogenetic trees based on the collective helical regions in the sequence alignment.

### Analyzing compensatory base changes

Compensatory base changes (CBC) occur when both nucleotides of a paired site mutate while the pairing itself stays stable. CBC analysis is important in detecting species that are discriminated by their sexual incompatibility [27-30]. We provide an easy-to-use CBC analysis mechanism with the ability to calculate CBC matrices on the current sequence and secondary structure based alignment. The numbers in the CBC matrix are the counts of compensatory base changes in a pairwise sequence structure alignment, which are naturally given in the considered multiple sequence structure alignment. A CBC-window in 4SALE (Fig. 2c) allows to select CBC counts between two sequences and highlights directly all CBCs within the alignment, giving an overview of all CBCs in the aligned sequences.

### Output & connection to other tools

For further analyses we provide several output formats. Calculated CBC matrices can be saved as comma/tab seperated values to be used in CBCAnalyzer [27]. CBCTree (as implemented in CBCAnalyzer) can be used to calculate phylogenetic trees based on a CBC count matrix. At present, no program is available to handle alignment outputs that include sequences and their individual secondary structures. However, for viewing purposes and publication we support a MARNA-like [14] output. Sequence alignments optimized by structural information could, of course, be saved separately. For phylogenetic analyses here we support the PHYLIP [31,32] formats. Other tools that rely on multiple alignments are supported by FASTA.

## Discussion

4SALE is the first alignment editor which allows synchronous editing of sequences and their corresponding secondary structures. Since it is targeted on RNA sequence alignment and editing it contains many features using the secondary structure information, e.g., the secondary structure inspector. All current standard alignment editors can handle secondary structures as character sequence only.

By using standard greedy protein alignment algorithms we inherit their time efficiency. In contrast to, e.g., MARNA [14] or RNAforester [15], the time complexity of calculation grows not rapidly with large files. We present a completely new approach using nucleotides and every single secondary structure for building and improving RNA sequence alignments in comparison to others, which just take the consensus structure information.

The structure output converted to Vienna style DotBracket can be created from any desired RNA folding program, e.g., RNAfold, Mfold [33] or RNAStructure [34]. It is then aligned by using a suitable substitution matrix, which in our case is based on information of the ITS2 Database.

Due to the natural limitation that two structures can be hidden in one sequence, in general only one will be considered by our approach.

A future version of 4SALE will integrate in addition to RNAforester [15] more real structural alignment methods as WebServices via the SOAP interface. Also secondary structure prediction algorithms as an alternative to RNAfold will be included. Furthermore, more visualizations like secondary structure drawings can be implemented.

## Conclusion

4SALE is easy to use and has a fast (<*O*(*N*^3^)) and good heuristic to globally align multiple RNA sequences and their associated secondary structures simultaneously.

## Availability and requirements

4SALE is freely available at . A JAVA virtual machine 5.0 is needed to run the application. Furthermore, for automatic sequence and structure based alignments a local installation of CLUSTAL W and/or internet connection for WebService based alignments is required.

## List of abbreviations used

CBC: compensatory base change

ITS2: internal transcribed spacer 2

## Authors' contributions

MW conceived the study. TM and MW provided the alignment algorithm. TM estimated the sequence and secondary structure substitution model and its associated score matrix. Architecture, implementation and graphical design by PS. MW, PS and TM drafted the manuscript. MW, TM, JS and TD participated in study design and coordination. All authors read and approved the final version of the manuscript.
